# The time course of the onset and recovery of axial length changes in response to imposed defocus

**DOI:** 10.1038/s41598-020-65151-5

**Published:** 2020-05-20

**Authors:** Samaneh Delshad, Michael J. Collins, Scott A. Read, Stephen J. Vincent

**Affiliations:** Queensland University of Technology, Centre for Vision and Eye Research, School of Optometry and Vision Science, Institute of Health and Biomedical Innovation, Kelvin Grove, Queensland Australia

**Keywords:** Anatomy, Medical research

## Abstract

The human eye is capable of responding to the presence of blur by changing its axial length, so that the retina moves towards the defocused image plane. We measured how quickly the eye length changed in response to both myopic and hyperopic defocus and how quickly the eye length changed when the defocus was removed. Axial length was measured at baseline and every 10 minutes during 1 hour of exposure to monocular defocus (right eye) with the left eye optimally corrected for two defocus conditions (+3 D and −3 D) and a control condition. Recovery was measured for 20 minutes after blur removal. A rapid increase in axial length was observed after exposure (~2 minutes) to hyperopic defocus (+7 ± 5 μm, p < 0.001) while the reduction in axial length with myopic defocus was slower and only statistically significant after 40 minutes (−8 ± 9 μm, p = 0.017). The eye length also recovered toward baseline levels during clear vision more rapidly following hyperopic than myopic defocus (p < 0.0001). These findings provide evidence that the human eye is able to detect and respond to the presence and sign of blur within minutes.

## Introduction

In a wide range of species, the quality of visual experience influences the axial growth of the eye during early life^1^. Depriving an eye of form vision by lid suture^2,3^ or by wearing translucent goggles or diffusers^4–6^ causes excessive eye growth and the development of myopia. Similarly, exposure of the eye to positive or negative optical defocus^7–12^ alters eye growth in a predictable way in order to compensate for the amount of imposed retinal blur. Visually guided eye growth begins with short-term changes in choroidal thickness which are followed by longer-term changes in eye growth^13–18^. The net result of these two mechanisms is a movement of the retina towards the defocused image plane to reduce the amount of imposed blur.

Animal studies investigating the time course of the eye’s response to blur have shown a rapid onset of choroidal thickness change, with only a few minutes of blur exposure required for the eye to discern the sign of blur and to elicit an appropriate directional choroidal response to minimize the amount of imposed blur^13–15^. The temporal properties of these compensatory changes are documented to vary according to the type of defocus imposed, with changes associated with myopic defocus being more enduring, suggesting that myopic defocus may produce stronger compensatory signals than hyperopic defocus^14,16^.

In human eyes, the response to short-term imposed defocus has also been investigated^17–20^. Exposing the eyes of children and young adults to short periods (1 or 2 hours) of monocular myopic and hyperopic defocus leads to small but statistically significant bi-directional changes in axial length (measured from the anterior cornea to the retinal pigment epithelium) and choroidal thickness^17,20,21^. In a recent study of presbyopic adults, significant bi-directional changes in choroidal thickness after 1 hour of imposed myopic and hyperopic defocus were also reported^22^. A 12-hour period of monocular myopic and hyperopic defocus has also been shown to alter the normal diurnal variations of both axial length and choroidal thickness in young adults^18,19^. Given that no significant change in anterior eye biometry has been observed during short-term imposed defocus, the changes in axial length with defocus have been primarily attributed to rapid changes in choroidal thickness^17,21,23^. Whilst these findings collectively imply that the human eye is able to discern the sign of defocus and make changes in the thickness of the choroid and hence axial length, details concerning how quickly this occurs, how the eye responds to defocus over time, and the time course of the decay of the eye’s response to defocus following the cessation of blur exposure are not well understood.

In this study we investigated the time course of the axial length response to 60 minute episodes of continuous myopic and hyperopic defocus, testing the hypothesis that the human eye, similar to those of other animals^17,18^, would be able to detect and respond to defocus within minutes of exposure to blur. We further assessed the persistence of axial length changes following the cessation of myopic and hyperopic defocus, during a period of clear vision, hypothesizing that the response to myopic defocus would be more enduring than the response to hyperopic defocus. Given that the visual system is also known to compensate for optical defocus through a gradual improvement in defocused visual acuity (VA) over time (blur adaptation)^24–28^, we also examined the association between the time course of changes in defocused VA and axial length during exposure to myopic defocus.

## Methods

Twenty-six young adults (14 females, mean age ± SD, 23.6 ± 3.7 years) were recruited. Prior to the experiment, each subject underwent a screening to ensure good ocular health, normal binocular vision and accommodation function, and to ascertain their refractive status. Refractive error was determined by non-cycloplegic subjective refraction using standard procedures. No subjects exhibited anisometropia of more than 0.50 DS or astigmatism of more than −0.75 DC. All subjects had up to date habitual spectacle prescriptions, and none were under myopia control treatment. No soft or rigid gas permeable contact lens wearers were included in this study to avoid potential changes in biometry associated with contact lens wear^29^. The Queensland University of Technology human research ethics committee approved the study. Written informed consent was obtained from each subject and the study adhered to the tenets of the Declaration of Helsinki.

This study involved a protocol investigating the short-term (60 minutes) influence of three different levels of imposed monocular defocus (+3 DS, −3 DS and 0 DS) on axial length, assessed before, during and after exposure to each defocus condition. To ensure diurnal variations of axial length did not confound the effects of defocus^30,31^, measurements were taken at a similar time of day for each defocus condition (between 8:00 am and 2:00 pm), and at least 2 hours after each subject’s reported time of waking. To prevent prior visual tasks (e.g. high accommodation demands) confounding the measurements of axial length^32–34^, before each measurement session, a “washout period” was implemented during which each subject binocularly viewed a television at a distance of 6 m with their optimal sphero-cylinder distance refractive correction in a trial frame. Subsequent to the “washout period”, the baseline measurement of axial length from the right eye was taken, and then a 60-minute “defocus period” was commenced.

During the “defocus period”, subjects were monocularly (right eye only) exposed to either a + 3 DS or a −3 DS defocus lens over their optimal distance correction, with their fellow left eye optimally corrected to maintain a relaxed state of distance accommodation. This monocular defocus paradigm has been implemented previously in several studies^17–19,21^. Following the “defocus period”, the defocus lens before the right eye was removed, and repeated measurements of axial length were carried out for a further 20 minutes, during the “recovery period” with a controlled 6 m distance viewing task. As a control condition, all of the experimental procedures were repeated with no blur (i.e. both eyes were optimally corrected for the duration of the control condition). For each subject, each test condition (+3 DS, −3 DS, and 0 DS) was conducted on a separate day, and the order of the three defocus conditions was randomized.

The Lenstar optical biometer (LS 900, Haag Streit AG, Koeniz, Switzerland) was used to measure axial length (the distance from the anterior corneal surface to the retinal pigment epithelium). On each measurement day, the axial length was measured at baseline (prior to introducing defocus), and then every 10 minutes during 60 minutes of imposed monocular defocus, with the initial measurement taking place after 2 minutes of exposure to defocus. Recovery of defocus-mediated changes in axial length was also assessed at 5 minute intervals during the 20-minute “recovery period”, with the initial measurement taking place after 2 minutes of exposure to clear vision. At each measurement time point, five repeated measures of axial length were obtained from the right eye (defocused eye).

**Figure 1:**
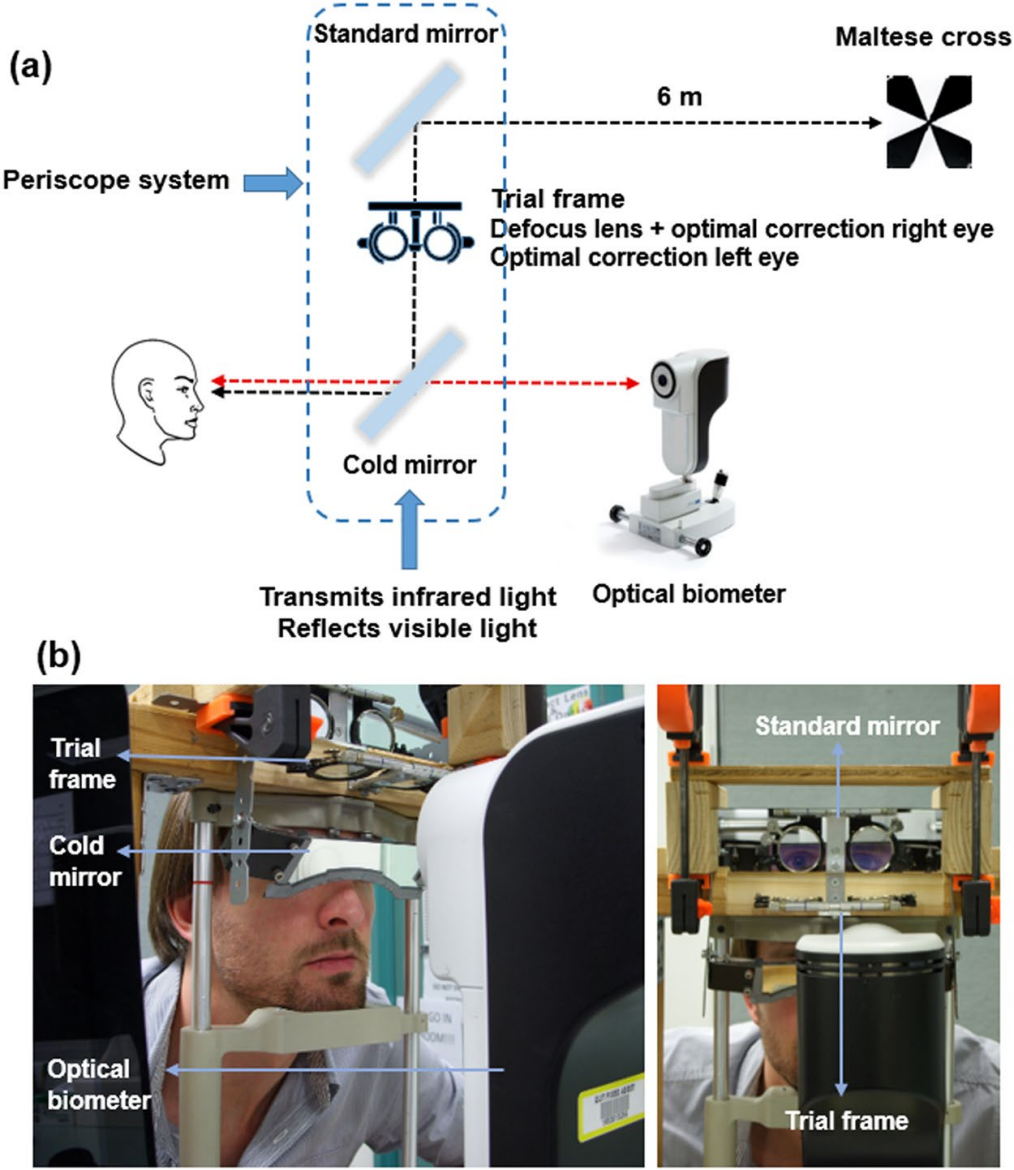
A schematic diagram (**a**) and photographs (from an oblique angle on the left and en face on right) of the experimental set-up (**b**). The binocular periscope system provides binocular viewing of the distance target (movie or Maltese cross) while allowing simultaneous Lenstar LS 900 measurements of the axial length.

To provide constant exposure to defocus and to control for accommodation during the measurements of axial length, a binocular periscope system attached to the biometer was used (Fig. 1). The periscopic view of an external target (high contrast Maltese cross displayed at 6 m distance on a TV screen) was provided for both eyes by adjusting the system, and once the centre of the Maltese cross was superimposed with the centre of the internal fixation target of the biometer (red fixation light), the subjects were asked to look at the centre of the Maltese cross and the measurements were taken. When using the periscope system, the subject’s vertex distance corrected sphero-cylinder distance refraction was placed in a trial frame before each eye and the additional defocus (equivalent to +3 DS or −3 DS at the corneal plane) was placed before the tested eye (right eye).

With the cold mirror placed in front of the eye, there was a reduction in the signal intensity arising from the anterior eye parameters and the Lenstar data was unable to provide anterior eye biometry in all subjects for all time points. To investigate whether the anterior eye biometry data were affected by the presence of defocus, for the subset of subjects where lens thickness (LT) and anterior chamber depth (ACD) data were available, analyses of changes in anterior eye biometry after 60 minutes of exposure to defocus were carried out. The number of subjects where LT was available during continuous myopic defocus was 10, LT during continuous hyperopic defocus was 12, LT during the control condition was 12, ACD during continuous myopic defocus was 13, ACD during continuous hyperopic defocus 13, and ACD during the control condition was 12.

Monocular VA was obtained from the right eye (defocused eye) of all subjects using the Early Treatment Diabetic Retinopathy Study (ETDRS) charts (Precision Vision, Vistakon Logarithmic Visual Acuity Charts, 9 series). Visual acuity was scored as the total number of letters read correctly and recorded in logMAR. The test was terminated when three or more letters per line was read incorrectly^35^. The VA was assessed at baseline, then at 10 minute intervals during 60 minutes of myopic defocus, with the initial measurement taking place after approximately 2 minutes of exposure to myopic defocus, and then following removal of the myopic defocus at the beginning of the recovery period. The persistence of defocused VA changes was also evaluated by reintroducing an equal amount of defocus (+3 DS) for a single measurement at the end of the 20 minute recovery period. For each VA measurement, the 9 ETDRS charts were randomized to reduce the potential for learning effects. To further ensure no learning effect had taken place, the changes in VA on the “control day” with continuous optimal correction were also assessed. Repeated measurements of axial length, and VA (or defocused VA) at baseline and during the “defocus period” and the “recovery period” were taken under the same experimental conditions and in a fixed ordered sequence, with axial length measured first, followed by VA.

The average of five repeated measures of axial length at each time point during each defocus condition were analysed for each subject. The Shapiro-Wilk test was used to confirm that the axial length and VA data were normally distributed. Axial length data from the defocus and the recovery periods were each analysed using a repeated measures analysis of variance (ANOVA) with two within-subjects factors of time and type of defocus (myopic defocus, hyperopic defocus or control). Visual acuity data were analysed using a repeated measures ANOVA with two within-subjects factors of time and type of defocus (myopic defocus or control). If significant main effects or interactions were found (p < 0.05), post hoc tests with Bonferroni correction were then conducted. The effects of defocus on anterior eye biometry data (LT and ACD) were assessed using Bonferroni adjusted paired t-test on the difference in measurements from baseline to the end of 60 minutes exposure to defocus.

In order to assess the possible associations between the changes in axial length and changes in defocused VA during the defocus and recovery periods, an analysis of covariance (ANCOVA) was carried out, using the method of Bland and Altman for calculating the correlation coefficient with repeated observations^36^. All statistical analyses were performed using SPSS for Windows software (version 21.0, SPSS Inc.).

## Results

### Axial length

**Figure 2:**
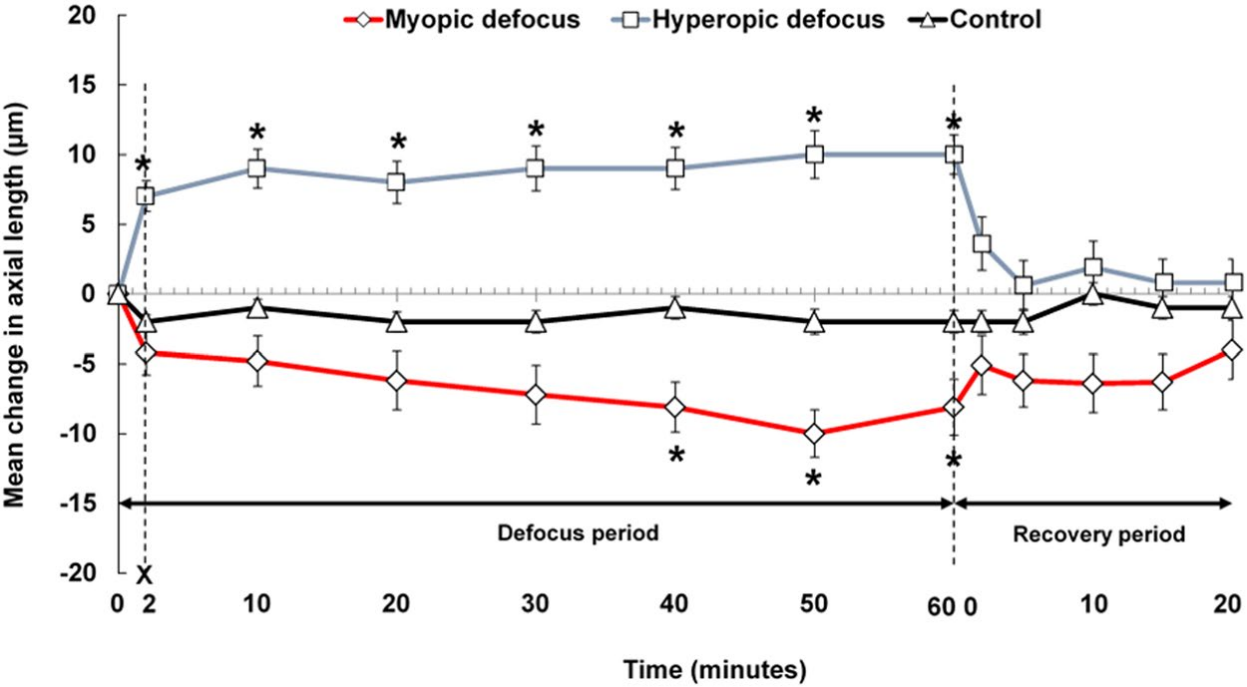
Mean change in axial length from baseline, during 60 minutes of monocular defocus (Defocus period) and 20 minutes of clear vision (Recovery period) for three defocus conditions (+3 D myopic, − 3 D hyperopic and optimal correction control). Error bars represent ± standard error of the mean. Asterisks (*) indicates a significant difference from the baseline axial length at 0 minutes (p < 0.05).

A significant within-subjects effect of type of defocus was observed for axial length measures during the defocus period (p = 0.006). The interaction between time and type of defocus was also significant (p < 0.0001). Figure 2 illustrates the group mean changes in axial length during the defocus and recovery periods across the three defocus conditions for all subjects.

Approximately 2 minutes after beginning exposure to hyperopic defocus, the group mean axial length increased significantly by +7 ± 5 μm (p < 0.001). Following this initial rapid response, axial length remained relatively stable over the next hour and was significantly longer than the baseline measurement at all subsequent time points (all p < 0.001). The maximum ocular elongation was observed after 50 minutes of exposure to hyperopic defocus, with a mean axial elongation of +10 ± 8 μm (p < 0.001).

The first statistically significant reduction in axial length occurred after 40 minutes of exposure to myopic defocus, with a mean reduction of −8 ± 9 μm (p = 0.017). This change peaked shortly after, reaching a maximum axial length reduction of −10 ± 8 μm at 50 minutes (p = 0.001). The eye then remained significantly shorter than the baseline axial length until the end of the myopic defocus period (mean difference of −8 ± 10 μm, p = 0.037) (Fig. 2). Axial length remained stable throughout the control condition with no significant differences observed between the baseline axial length and any of the subsequent axial length measures (all p > 0.05) (Fig. 2).

Repeated measures ANOVA revealed a significant interaction between the type of defocus and time for axial length measures during the recovery period (p = 0.003). Approximately 2 minutes after the removal of the myopic defocus, the shortened eye elongated by 37%. The eye then remained relatively stable over the next 20 minutes, recovering towards the baseline level by almost 50% after 20 minutes, but was still −4 ± 10 μm shorter than the baseline axial length (p > 0.05) (Fig. 2).

Approximately 2 minutes after the removal of hyperopic defocus, the elongated eye recovered significantly by 63% (p = 0.010). The eye then continued to shorten rapidly over the next 20 minutes, recovering by 91% at the end of 20 minutes (mean difference of +1 ± 10 μm relative to the baseline, p > 0.05). During the control condition, there was no significant difference between baseline axial length and any subsequent axial length measures during the recovery period (all p > 0.05) (Fig. 2).

To further compare the pattern of changes in axial length when the eye was recovering from the myopic and hyperopic defocus conditions, a linear mixed-model analysis was used to fit a regression line to the axial length recovery data of each defocus condition and the slope and intercept of the two regression lines was compared. A highly significant difference was observed between the slopes of the recovery of axial length between myopic and hyperopic defocus conditions during 20 minutes of clear vision (slope ß, myopic = 0.109 vs hyperopic defocus = −0.313, p < 0.001). The estimated time for complete recovery to the baseline axial length was then determined based on the intercept of the regression line. For hyperopic defocus, a return to the baseline axial length was estimated to occur after 21 minutes of exposure to clear vision, compared to 35 minutes for myopic defocus. Significant differences were observed in these intercepts of the complete recovery of axial length to the baseline values between the myopic and hyperopic defocus conditions (p < 0.001).

### Defocused visual acuity

**Figure 3:**
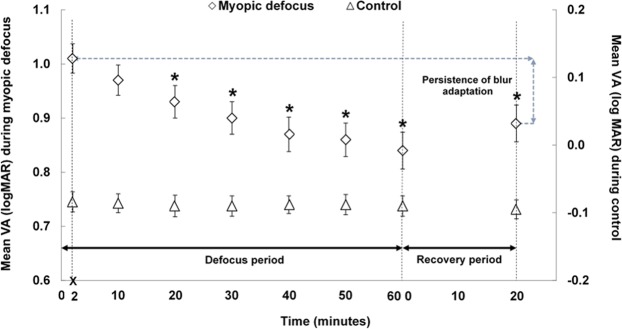
Group mean VA at each time point during 60 minutes of monocular defocus (Defocus period), and its persistence after 20 minutes of clear vision (Recovery period) during the myopic defocus and control conditions. Error bars represent ± standard error of the mean. Asterisks (*) indicates a significant mean difference from the initial defocused VA (p < 0.05).

A significant interaction was observed between the type of defocus and time for the defocused VA measures during the defocus period (p < 0.001) (Fig. 3). After the right eye was exposed to myopic defocus, baseline VA (−0.06 ± 0.07 logMAR) decreased significantly to 1.01 ± 0.13 logMAR (defocused VA) (p < 0.001). The defocused VA then improved gradually over time, reaching 0.93 ± 0.15 logMAR after approximately 20 minutes (p < 0.001); accounting for nearly 50% of the total defocused VA improvement. From then until the end of defocus period, the defocused VA continued to improve, but at a slower rate. The maximum blur adaptation effect was observed at the end of the defocus period, with a mean improvement of 0.16 ± 0.12 logMAR from the initial defocused VA (p < 0.001). When the defocus lens was reintroduced at the end of the 20 minute recovery period, the blur adapted VA showed 70% persistence, since the defocused VA was still 0.11 ± 0.10 logMAR better than the initial defocused VA measured at the beginning of the defocus period (p < 0.001). During the control condition, VA remained stable (all p > 0.05 from the baseline measurement) (Fig. 3).

### Association between axial length changes and defocused visual acuity

ANCOVA for repeated measures revealed a significant but weak positive association between the changes over time in axial length and defocused VA following exposure to myopic defocus (ß slope = 0.015, r^2^ = 0.055, p = 0.003). As axial length decreased over time in the presence of myopic defocus, the defocused VA value decreased (i.e. the defocused VA improved), with an improvement of 0.1 logMAR in defocused VA over time being associated with an axial length decrease of 5 μm. No significant association was found between the change in axial length and VA during recovery period or during the control condition.

There was no significant change in baseline LT and ACD after exposure to continuous myopic and hyperopic defocus (p > 0.05). The mean change in LT after exposure to continuous myopic defocus was +1 ± 15 µm, and −5 ± 12 µm after exposure to continuous hyperopic defocus. The mean change in ACD after exposure to continuous myopic defocus was −6 ± 9 µm, and −3 ± 10 µm after exposure to continuous hyperopic defocus. The changes in LT and ACD during the control condition were also not significant (+4 ± 13 µm and +5 ± 11 µm, respectively, p > 0.05).

## Discussion

In young adults, the eye appears capable of discerning the sign of defocus rapidly (within minutes of exposure to defocus) and making compensatory changes in its axial length, which would have the effect of moving the retina in the direction towards the defocused image plane. These findings are consistent with the general findings from previous reports in humans^21,23^ and animals^13–15^, where changes in axial length and choroidal thickness occur shortly after exposure to blur. We demonstrated that the speed with which the eye changes its axial length varies according to the sign of defocus, with the eye elongating faster during hyperopic defocus than it shortens during myopic defocus. Our study further suggests a slightly greater persistence of the effects of myopic defocus than hyperopic defocus on axial length during a recovery period of clear vision after defocus.

Previous studies in human adults have measured short-term axial length changes in response to defocus after 30 minutes and 1 hour^20,21^. With myopic defocus, there were axial length reductions of 9 μm after 30 minutes and 13 μm after 60 minutes, and with hyperopic defocus there were axial length elongations of 5 to 9 μm after 30 minutes, and 8 to 11 μm after 60 minutes^20,21^. Our findings from the current study are consistent with these previous reports in young adults, with an average decrease of −7 µm with myopic defocus and an average increase of +9 µm with hyperopic defocus after 30 minutes which then peaked at ±10 µm of change after 60 minutes.

By using a higher sampling frequency rate with measurements of axial length every 10 minutes (with the initial measurement occurring after only 2 minutes of blur exposure), we further showed that the axial length of the human eye can change in response to relatively brief periods of myopic and hyperopic defocus. Our results demonstrate that after ~2 minutes of exposure to hyperopic defocus; the human eye can discern the sign of blur and increase its axial length which moves the retina towards the defocused focal plane. The response to myopic defocus was slower, showing a significant compensatory shortening of axial length only after 40 minutes of continuous exposure. In a recent report, Chiang *et al*.^23^ demonstrated a temporal difference in the choroidal response to myopic and hyperopic defocus, with a faster choroidal thickening in response to myopic defocus (after 10 minutes) and a slower choroidal thinning in response hyperopic defocus (after 20–30 minutes). Given that the changes in axial length with defocus are expected to be initiated from the changes in the choroidal thickness (i.e. as the choroid thickens the distance to the retinal pigment epithelium {axial length} decreases and vice versa), a similar temporal pattern of response to defocus for axial length and choroidal thickness is expected. The exact reason for the discrepancy between our findings and findings from Chiang *et al*. is unclear but may be due to the ethnicity of the subjects in the Chiang *et al*. study which included only South-East Asian participants compared to our relatively ethnically diverse cohort which consisted of 50% Caucasian, 39% East Asian, and 11% Indian.

Chick models have also shown rapid responses to defocus, with either a similar time course of change in the rates of ocular elongation and choroidal thickness for positive and negative lenses^14^, or a faster time course of change for positive compared to negative lenses^13^. Zhu and Wallman reported that for both positive and negative lenses, about 1 to 4 minutes of exposure to defocus was sufficient to initiate the appropriate compensatory signals required for modulations of ocular elongation and choroidal thickness^14^. Another study in chicks has reported the required time to elicit appropriate compensatory changes in the choroid was 10 minutes for myopic defocus and 60 minutes for hyperopic defocus^13^.

Following the cessation of defocus, the axial shortening effects of myopic defocus were slower to trend towards baseline levels than the hyperopic defocus effects. After 20 minutes of clear vision, on average the eye recovered by 50% from myopic defocus while it recovered by 90% from hyperopic defocus. A longer lasting effect for myopic than hyperopic defocus has also been reported previously in chicks. For instance, 2 to 3 hours of unrestricted vision per day in eyes wearing negative lenses reduced eye elongation by almost 95%, whereas in eyes wearing positive lenses, eye shortening was reduced by only 10% after three hours of daily unrestricted vision^16^. Also, chicks exposed to only three hours of myopic defocus per day and unrestricted vision for the remainder of the day (9 hours), still exhibited a significant hyperopic shift^16^. In another study, when various episodes of darkness were imposed between episodes of defocus, a 50% decay in the axial length effects of myopic defocus occurred after 24 hours while for hyperopic defocus, it occurred after 24 minutes^14^.

The method through which the human eye is able to detect the sign of defocus is unknown; however, a trial-and-error mechanism of blur identification has been previously suggested^37^. If such a mechanism contributes to blur identification in the human eye, the magnitude of change in the refraction as a result of defocus-induced changes in axial length should be greater than the eye’s depth of focus. In this study, we observed a maximum axial length change of 10 μm with myopic and hyperopic defocus which corresponds to only a 0.03 D change in the ocular refraction. This amount of change in refraction is small compared to the depth of focus of the eye, which ranges between ~0.15 D to 0.27 D (for pupil diameters of between 5–6 mm)^38–40^. Therefore, the possibility of a trial and error mechanism of blur identification related to axial length changes seems unlikely. Alternatively, other methods such as contrast cues from contrast adaptation (changes in contrast sensitivity at different spatial frequencies)^41–44^, colour cues from chromatic aberration^45–47^, or optical vergence cues from image defocus^48^ could be used by the human eye to decode the sign of blur.

The rapid changes in axial length in response to defocus observed in our study most likely occurred at least partly through rapid modulations in the thickness of the choroid, but the underlying mechanism is not known^17–19,21,23,49^. The defocus-mediated changes in the thickness of the choroid are reported to occur within minutes after exposure to myopic and hyperopic defocus in both animal^13,14^ and human eyes^23^.

Similar to the previous reports in human eyes^18,19,21^, we found no significant effects of defocus on lens thickness or anterior chamber depth in our study. Given that our study protocol involved exposure to monocular hyperopic defocus (with the fellow eye open and optimally corrected), it was important to ensure that the subjects did not accommodate through the hyperopic defocus lens, which would have minimized its potential effects in the tested eye and induced myopic defocus in the fellow eye. The lack of a significant change observed in both lens thickness and anterior chamber depth with defocus suggests that during monocular hyperopic defocus exposure, the accommodation state of the defocused eye remained relatively unchanged and the clear fellow eye guided the accommodation response in both eyes.

Our investigation of the short term axial length response to blur might have implications for understanding the mechanisms underlying eye growth in humans. For instance, it has been proposed that transient exposure to hyperopic defocus associated with near activities (e.g. due to a lag of accommodation)^50,51^, ocular aberrations^52^ and peripheral defocus^53^ might predispose the eye to myopia. We found the axial length effects of hyperopic blur to quickly subside during short exposure to clear vision, with 2 minutes of clear vision being sufficient to significantly reduce the axial elongation effects of hyperopic defocus. If ocular changes resulting from short-term exposure to hyperopic defocus are associated with longer-term refractive error development in human eyes, then imposing brief exposures to clear vision (e.g. taking frequent breaks by looking at far objects in between near activities) may counterbalance the induced myopigenic hyperopic blur stimulus. Further, incorporating myopic defocus into bifocal or multifocal lenses as an optical intervention to retard myopia progression in school children has shown modest treatment benefits^54–58^. We found a continuous exposure of a minimum of 40 minutes was required for myopic defocus to have a significant effect on axial length reduction in human eyes.

Blur adaptation effects were also measurable within minutes after beginning exposure to defocus, as reported previously^28,59^. The magnitude of improvement in the defocused VA over 60 minutes was 0.16 logMAR, which is consistent with the magnitude previously reported in young adults^28,60^. A neural sensitivity gain adjustment in different spatial frequency channels of the visual system has been proposed as a possible underlying mechanism that may mediate this response^24,25,61,62^. We found that nearly 60% of the VA improvement observed after one hour of myopic defocus remained after 20 minutes of clear vision. The durable nature of blur adaptation effects on defocused VA has previously been reported^63,64^.

In this study we tested whether the defocus-mediated changes in VA and axial length are strongly associated, which could indicate a common underlying mechanism. That is, the level of adaptation in VA is providing cues to the level (not sign) of image defocus which in turn guides the changes in axial length to move the retina towards the defocused image plane^15,41,44^. Both axial length and defocused VA changed rapidly following exposure to myopic defocus. We investigated the possible association between the temporal changes in defocused VA and axial length during myopic defocus and observed a weak but statistically significant, positive association, with a 0.1 logMAR improvement in defocused VA over time being associated with a 5 µm of change in axial length. Since a 5 μm of change in axial length is approximately equivalent to a 0.012 D change in ocular refraction^65^, this amount of change is too small to influence the improvement in VA. Further, the weak association between the defocus-mediated changes in axial length and VA does not imply a causal link between the two changes in the eye.

Our findings from these investigations are limited to the level of defocus used and the durations of the periods of defocus and clear vision. Future studies repeating these experiments at different ages, and utilizing different levels and durations of defocus and clear vision may expand our knowledge of the role of defocus in modulating the eye growth. Further, the temporal properties of the choroidal response to defocus blur were not investigated in this study. Given the important role of the choroid in modulating the eye growth^49,66^, the temporal characteristics of its changes to blur need to be investigated in detail.

In summary, we have shown for the first time that in young human adults, the eye is able to discern the sign of defocus within minutes after exposure to blur and make changes in its axial length in a direction to reduce the amount of retinal blur. The human eye elongated faster during hyperopic defocus than it shortened during myopic defocus and, similar to other animal models, the ocular response to myopic defocus was found to be more enduring than hyperopic defocus after removing the defocus stimulus. Whilst these findings improve our understanding of the temporal properties of the eye’s response to defocus blur, further research is required to understand the underlying mechanisms that mediate these responses to defocus blur.

## Data Availability

The datasets generated and analysed during this study are available from the corresponding author on reasonable request.
